# Gender differences in brain structure and resting-state functional connectivity related to narcissistic personality

**DOI:** 10.1038/srep10924

**Published:** 2015-06-25

**Authors:** Wenjing Yang, Lingli Cun, Xue Du, Junyi Yang, Yanqiu Wang, Dongtao Wei, Qinglin Zhang, Jiang Qiu

**Affiliations:** 1 Key Laboratory of Cognition and Personality (SWU), Ministry of Education, Chongqing 400715, P. R. China; 2 School of Psychology, Southwest University, Chongqing 400715, P. R. China

## Abstract

Although cognitive and personality studies have observed gender differences in narcissism, the neural bases of these differences remain unknown. The current study combined the voxel-based morphometry and resting state functional connectivity (rsFC) analyses to explore the sex-specific neural basis of narcissistic personality. The VBM results showed that the relationship between narcissistic personality and regional gray matter volume (rGMV) differed between sexes. Narcissistic scores had a significant positive correlation with the rGMV of the right SPL in females, but not in males. Further analyses were conducted to investigate the sex-specific relationship between rsFC and narcissism, using right SPL/frontal eye fields (FEF) as the seed regions (key nodes of the dorsal attention network, DAN). Interestingly, decreased anticorrelations between the right SPL/FEF and areas of the precuneus and middle frontal gyrus (key nodes of the the default mode network, DMN) were associated with higher narcissistic personality scores in males, whereas females showed the opposite tendency. The findings indicate that gender differences in narcissism may be associated with differences in the intrinsic and dynamic interplay between the internally-directed DMN and the externally-directed TPN. Morphometry and functional connectivity analyses can enhance our understanding of the neural basis of sex-specific narcissism.

Narcissism, which is well documented in the literature, is often described as a personality characteristic involving arrogance, a feeling of entitlement, and the willingness to exploit others, and it is correlated with dominance and aggression^1^,^2^,^3^. It is a central personality construct in both research and clinical contexts. In the field of social-personality, narcissism is thought to be a trait that is normally distributed in the population^3^. This quality has significant implications for thinking, feeling, and behaving. Thus, a substantial amount of research in psychoanalysis and self psychology has been done to examine the concept of narcissism^2^,^3^,^4^,^5^,^6^. However, research on narcissism is fraught with controversy.

The issue of gender differences in narcissism is a particularly interesting and controversial topic. Although some studies have reported no gender differences in narcissism^2^,^4^, A more consistent view is that males typically score somewhat higher on the Narcissistic Personality Inventory (NPI) than females do^5^,^6^,^7^,^8^,^9^. Other research also suggests that the dimensions of the construct of narcissism differ between men and women. For instance, Tschanz *et al.*^9^ found that the dimension of exploitativeness/entitlement had lower correlations with other narcissistic dimensions in women than in men. In addition, some studies have found evidence for greater use of “stereotypical narcissistic” behaviours by men. For example, male narcissists are more likely than female narcissists to employ heightened self-handicapping^10^, and to show self-enhancement when modesty was called for^11^. In sum, narcissism in males has been linked to striving for authority, feelings of entitlement and power^12^,^13^; self-handicapping^10^; heightened aggressive behaviour following criticism^14^; and a preference for interpersonal competition^15^. In contrast, female narcissists seek affiliation with desirable others^11^; appraise themselves more positively in terms of their physical appearance, physical health, and sexuality^16^,^17^; and are likely to be more socially anxious than males^18^. Narcissistic males are likely to be excessive in their efforts to assert superiority, whereas the goals of narcissistic females are achieved through more subtle, indirect, and affiliative means that are consistent with gender expectations.

Although the concept of narcissism has been much studied in psychology, its neural underpinnings remain unclear. Indeed, there has been a debate on whether specific brain substrates are associated with narcissism. A large body of research has linked narcissism to different characteristics of the self, such as grandiosity, the need for admiration, a lack of emotional empathy, and alexithymia^3^. Research suggests that grandiosity and the need for admiration stem from the same underlying roots, that is, distorted self-views and self-enhancement^11^,^19^. Some studies have shown that the frontal lobes, including the middle frontal gyrus (MFG), medial prefrontal cortex (MPF), orbitofrontal cortex (OFC)^20^, precuneus (PC), and anterior insula (AI) are involved in self-enhancement and self-evaluation^20^,^21^. Research also suggests that lack of empathy, or lack of interest in warm and caring interpersonal relationships, is the third feature of narcissism^3^,^22^, and that the AI^23^, the right dorsolateral prefrontal cortex (DLPFC), and the right posterior cingulated cortex (PCC)^24^ might be involved in lack of empathy.

Previous research has suggested that the term narcissism might describe different phenomena in the two genders, which raises the possibility that narcissism in females and males might have different neural substrates. Studies already have demonstrated that narcissistic males are more likely than females to be excessive in their efforts to assert superiority. Compared to female narcissists, male narcissists also are: more prone to interpersonal competition and exploitativeness; more likely to employ heighted self-handicapping and have a sense of entitlement; and less likely to be empathic^9^,^10^,^11^. Females, on the other hand, are more likely to enhance their social power through means such as seeking affiliations with glamorous others^11^. Hence, we hypothesized that sex-specific neural substrates in narcissism might be observed for the three basic dimensions of narcissism itself. Specifically, we expected to observe a gender difference in the frontal lobes, PC, or AI. The study combined structural imaging and resting-state functional connectivity (rsFC) analysis to explore the sex-specific neural basis of narcissism. The examination of anatomical features using structural imaging can be particularly efficacious for investigations of stable individual personality traits (including narcissism)^25^. In addition, rsFC can reflect functional relationships between different brain regions based on temporal correlations in blood oxygen level-dependent (BOLD) signals during rest^26^. To the best of our knowledge, no study has explored the sex-specific neural mechanisms of narcissism. We predicted that the combination of the voxel-based morphometry and functional connectivity analyses would enhance our understanding of the neural basis of sex-specific narcissism.

## Results

### Descriptive statistics

The demographic data and behavioural results are shown in Table 1. Males showed significantly higher total NPI scores than females (*P* < 0.05, *t* = 3.26, two-tailed *t*-test), especially on the subscales of authority, superiority, entitlement, and self-sufficiency (*P* < 0.05). These results are consistent with those of previous studies^1^,^5^. The kurtosis (−0.247) and skewness (0.412) of the NPI variable are acceptable for an assumption of normality (this ranges between −1 and +1)^27^. In addition, the internal reliability of the scale was satisfactory (Cronbach’s *α* = 0.86). Furthermore, the NPI score was significantly correlated with the SES score (*r* = 0.38, *P* < 0.05) for all subjects.

### Interaction effect of sex and narcissism on regional gray matter volume (rGMV)

**Figure 1:**
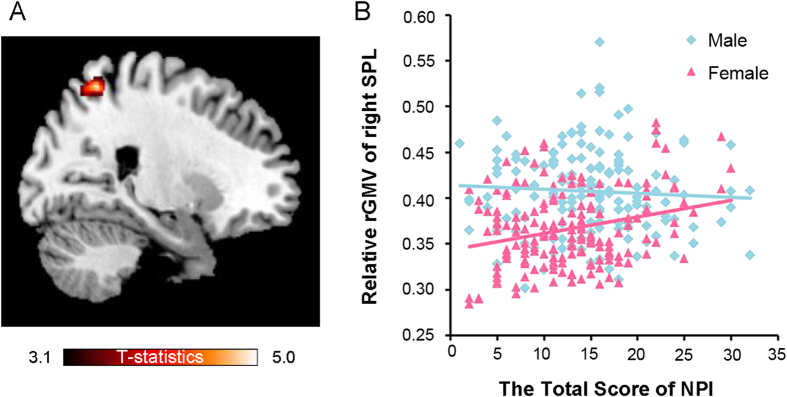
Voxel-based morphometry results (**A**) Sex modulates the effect of narcissism on gray matter in the right SPL (Results were shown for *P* < 0.05, corrected for multiple comparisons at the cluster-level, with an underlying voxel-level of *P* < 0.001, uncorrected; the same voxel-level thresholds as are used in Figs. 2 and 3). (**B**) Scatter plots of the relationship between NPI scores and rGMV values at the peak voxel of the right SPL (*x, y, z* = 23, −56, 60). The rGMV of the right SPL has a significant positive correlation with the NPI scores of females (*r* = 0.27, *P* < 0.05), whereas it does not have a significant correlation with the NPI scores of males (*r* = −0.06; *P* > 0.05).

A voxel-wise analysis of covariance (ANCOVA) revealed an interaction effect of sex and total NPI score on rGMV in the right SPL region: MNI coordinates: *x, y, z* = 23, −56, 60, *t* = 5.05, *P* < 0.05, corrected for multiple comparisons using the voxel-level family-wise error (FWE) at the whole-brain level; see Fig. 1(A) and Table 2. In particular NPI scores had a significant positive correlation with the rGMV of the right SPL in females (*r* = 0.27, *P* < 0.05), but not in males (*r* = −0.06; *P *> 0.05), as seen in Fig. 1(B). We also examined sex differences for each dimension of the NPI and rGMV; however, no significant associations were observed (*P* > 0.05, FWE corrected). Nevertheless, there was a tendency for each NPI dimension to be associated with rGMV in the right SPL region. The statistical values and coordinates of the peak voxel in this region were as follows: *x, y, z* = 23, −54, 60, *t* = 3.18 for authority; *x, y, z* = 24, −54, 59, *t* = 3.75 for exhibitionism; *x, y, z* = 24, −54, 62, *t* = 3.32 for superiority; *x, y, z* = 26, −48, 59, *t* = 3.17 for en*t*itlement; *x, y, z* = 20, −59, 59, *t* = 3.05; for exploitativeness; *x, y, z* = 23, −54, 60, *t* = 3.78 for self-sufficiency; and *x, y, z* = 21, −57, 60, *t* = 3.79 for vanity. The results of the seven NPI subscales were so similar that they may not provide any additional meaningfully information about sex differences.

### RsFC with seed regions

**Figure 2:**
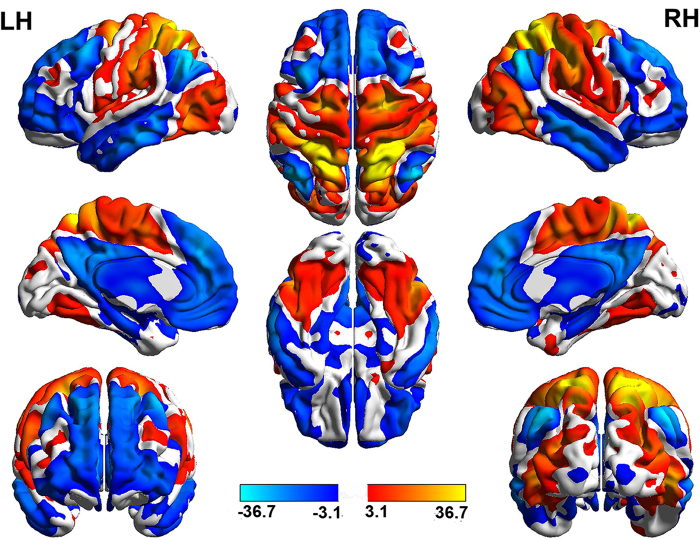
Regions with positive or negative rsFC with seed regions (right SPL). The results are shown for *P* < 0.001, uncorrected. Colour bars represent T-scores. The orange regions represent positive rsFC with the right SPL (seed), and the blue regions represent negative rsFC with the right SPL (seed).

The regions with positive or negative rsFC with seed regions are shown in Fig. 2. The right SPL showed positive rsFC with a broad network, including the middle temporal region (MT^+^), precentral sulcus, and frontal eye fields (FEF). Based on the previous studies, all these regions were key nodes of task positive regions^28^. Moreover, the MPF, PCC/PC and lateral parietal cortex (LP) showed negative rsFC with the right SPL. These regions were key nodes of task negative regions by visually checked^28^.

### Interaction effect of sex and narcissism on rsFC

**Figure 3:**
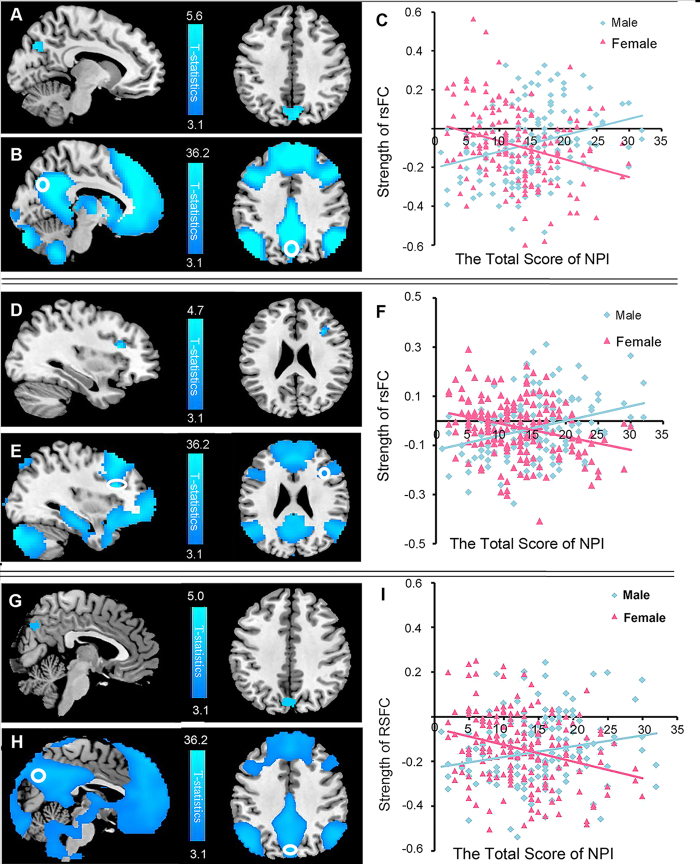
Regions showing the interaction effects of NPI score and sex on the strength of rsFC in the right SPL/FEF (seeds). Results are shown for *P* < 0.05, corrected for multiple comparisons at the cluster-level with an underlying voxel-level of *P* < 0.001, uncorrected. (**A)** A significant, negative interaction effect of sex and narcissism was found on the strength of rsFC between the right SPL (seed) and the right PC. (**B)** The position of the PC (marked with a white circle) pertains to the negative functional connectivity map of SPL (seed). (**C)** Scatter plots of the relationships between NPI scores and the rsFC strength between the right SPL and right PC (peak voxel values) (females: *r* = −0.30, *P* < 0.05; males: *r* = 0.30, *P* < 0.05). (**D)** A significant, negative interaction effect of sex and narcissism was found on the strength of rsFC between the right SPL (seed) and right MFG. (**E)** The position of the MFG (marked with a white circle) pertains to the negative functional connectivity map of the SPL (seed). (**F)** Scatter plots of the relationships between NPI scores and rsFC strength between the right SPL and right MFG (peak voxel values) (females: *r* = −0.28, *P* < 0.05; *r* = 0.32, *P* < 0.05). (**G)** The negative interaction effect of sex and narcissism on the strength of rsFC between the right FEF (seed) and the bilateral PC. (**H)** The position of the PC (marked with a white circle) pertains to associated with the negative functional connectivity map of the FEF (seed). (**I)** Scatter plots of the relationships between NPI scores and the rsFC strength between the right FEF and the bilateral PC (peak voxel values) (females: *r* = −0.28, *P* < 0.05; males: *r* = 0.20, *P* < 0.05).

ANCOVA was used to examine the interaction of sex and narcissism (controlling for age, SES, and mean framewise displacement) on the strength of rsFC with the right SPL [the seed region, one of the key nodes of dorsal attention network (DAN)]. Negative interaction effects were found for sex and narcissism scores on the strength of rsFC between the right SPL (the seed region) and the right PC (*x, y, z* = 6, −75, 39, *t* = 5.61, *P* < 0.001, corrected for multiple comparisons at voxel-level FWE at the whole-brain level – see Fig. 3(A) and Table 2. The left PC also exhibited the same tendency. Negative interaction effects was also found for sex and narcissism scores on the strength of rsFC between the right SPL (the seed region) and the right MFG (*x, y, z* = 33, 21, 21, *t* = 4.76, *P* < 0.05, corrected for multiple comparisons using the voxel-level FWE at the whole-brain level) – see Fig. 3(D) and Table 2. No positive interaction effects were found. The NPI scores of females were negatively correlated with rsFC strength between the right SPL and right PC (*r* = −0.30, *P* < 0.05), and between the right SPL and right MFG (*r* = −0.28, *P* < 0.05). However, these correlations we*r*e positive in males (*r* = 0.30, *P* < 0.05; *r* = 0.32, *P* < 0.05). A whole-brain ANCOVA was used to examine the relationship between each of the seven NPI dimensions and rsFC with the right SPL. The results were similar to the results for the total NPI score, but they were not significant.

In order to further validate our findings, we conducted analyses with some brain regions in the task positive network (TPN): the insula (MNI : −31, 21, −1; 31, 22, −2), the DLPFC (−50, 20, 34; 46, 14, 43), FEF (−25, −8, 50; 27, −8, 50), and the supplementary motor area (SMA, −8, 6, 55; 9, 10, 53). The first three seeds were taken from Vincent *et al΄s.* study^29^, while the last seed (the SMA) was taken from Corbetta *et al΄s.* study^30^. These regions were defined as a sphere with a 6-mm radius centered on the peak voxel. Interestingly, negative interaction effects were found for sex and narcissism on the strength of rsFC between the right FEF and bilateral PC (*x, y, z* = 3, −75, 39, *t* = 5.0; *x, y, z* = −9, −75, 33, *t* = 4.59, *P* < 0.001, FWE corrected) – see Fig. 3(G) and Table 2, and between the right insula and left mPFC (*x, y, z* = −12, 57, 15, *t* = 4.12, *P* < 0.001, uncorrected). In addition, a complementary analysis was conducted to ascertain the possible link between the structural and functional results. Because we only found the altered GMV in female participants, a correlation analysis was performed to investigate the relationship between altered GMV in the right SPL and the anticorrelations between the right SPL and right MFG in females. The results showed that there was a negative correlation between the GMV of the right SPL and the rsFC between the right SPL and right MFG (*r* = −0.17, *P* < 0.05). This results suggested that if the GMV of the right SPL was larger in female participants, the anticorrelation of the rsFC between the right SPL and the right MFG would be stronger.

An ANCOVA used to explore the interaction effect of sex and narcissism on the strength of rsFC in the DMN (seed regions: mPFC and PC/PCC) found no significant interaction effect on the strength of rsFC in the DMN.

Finally, in order to examine if our study was affected by sample specific variance and whether any other random split of the data might lead to as many interactions, the split half approach (internal replication approach) was used to validate our findings. First, our sample (150 males and 176 females) was randomly divided in half, and then VBM and rsFC analyses were conducted (see the Methods section). As expected, the first half of the sample (75 males and 88 females) exhibited an interaction of sex and total NPI score on rGMV in the right SPL region (MNI coordinate: *x, y, z* = 24, −51, 60, *t* = 4.14, *P* < 0.001, uncorrected). Negative interaction effects also were found for sex and narcissism on the strength of rsFC between the right SPL (the seed region) and the right PC (*x, y, z* = 6, −72, 39, *t* = 4.02, *P* < 0.001, uncorrected), and between the right SPL (the seed region) and the MFG (*x, y, z* = 33, 24, 18, *t* = 4.16, *P* < 0.001, uncorrected). In addition, the second half of the sample exhibited an interaction of sex and total NPI score on rGMV in the right SPL region (MNI coordinate: *x, y, z* = 20, −59, 60, *t* = 4.06, *P* < 0.001, uncorrected). A negative interaction effect of sex and narcissism also was found for the strength of rsFC between the right SPL (the seed region) and the bilateral PC (*x, y, z* = 6, −75, 39, *t* = 4.20; *x, y, z* = −6, −75, 39, *t *= 4.12, *P *< 0.001, uncorrected), and between the right SPL (the seed region) and the MFG (*x, y, z* = 33, 21, 20, *t* = 4.02, *P* < 0.001, uncorrected).

## Discussion

**Figure 4:**
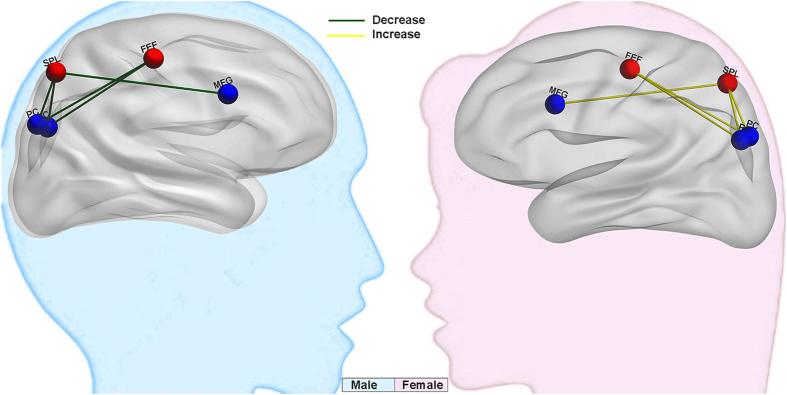
Resting state functional connectivity maps between DAN (SPL, FEF) and DMN (PC, MFG). Males showed decreased (the green line) anticorrelations between this rsFC and narcissism, whereas females showed the opposite tendency (increased anticorrelation, the yellow line).

This study combined the voxel-based morphometry and resting state functional connectivity (rsFC) analyses to explore the sex-specific neural basis of narcissism in a large sample (N = 326) of healthy adults. To the best of our knowledge, this is the first study of its kind. As expected, males reported significantly higher NPI scores than females, especially in the dimensions of authority and entitlement, which was consistent with previous findings^5^. Structural data showed that narcissism was positively correlated with the rGMV of the right SPL only in females. Furthermore, there were interaction effects of sex and narcissism on the strength of rsFC between SPL/FEF (key nodes of the DAN) and PC/MFG (key nodes of the DMN). Specifically, males showed decreased anticorrelations between these rsFCs and narcissism, whereas females showed the opposite tendency: increased anticorrelation between the SPL/FEF and PC/MFG (see Fig. 4). There was a negative correlation between the GMV of the SPL and an anticorrelation between the right SPL and right MFG in females. The present study provides the first empirical evidence of sex-specific neurological underpinnings for narcissistic personality. The following discussion addresses the implications of these findings.

Higher narcissism in females was associated with a larger rGMV of the right SPL, which might be associated with attention seeking, especially attention that concerns physical appearance. Previous studies have indicated that the SPL is one of the key nodes of the DAN, which has been linked to focused attention on external stimuli^31^,^32^. Specifically, studies have suggested that the SPL might be involved in seeking and selectively attending to significant extrapersonal stimuli^33^,^34^, and reconfiguring or shifting attention rather than maintaining attention^35^,^36^,^37^. Previous studies suggest that the social construction of the asymmetrical development of women and men in the period of early childhood may force females to meet their narcissistic goals through subtle, indirect, and affiliative means that conform to the expectations of their sex role^3^. Thus, they tend to pay more attention to external validation, seeking affiliation with glamorous others^6^,^10^,^11^. They appraise themselves more positively in terms of physical appearance, physical health, and sexuality^16^,^17^. Narcissism is associated with self-focused thoughts and behaviours, which reflect internally-directed cognition. This involves self-relevant information processing (self-related fantasies) and spontaneous cognition^38^,^39^,^40^. However, females also should have more extrospective processes to seek affliation with others and construct the grandiose self-view.

Most importantly, we found increased anticorrelations between the SPL/FEF and the PC/MFG in high scoring narcissistic females. The PC and mPFC are key nodes of the DMN, which is implicated in self-consciousness and self-related mental representations during rest 41,42,43. The DAN (belonging to the TPN) and SPL/FEF are engaged in cognitive processes associated with focused attention-demanding tasks, particularly those that involve representing external information when attempting to attain a goal^44^,^45^,^46^. In contrast, the DMN, PC, mPFC, and MFG are involved in internally focused tasks, including the retrieval of autobiographical memories and self-relevant processing^28^,^47^. The current study showed that the TPN (especially DAN) has an anticorrelation with the DMN. As early as 2005, Fox *et al.* noted that the anticorrelation between the two intrinsic networks (the TPN and the task-negative network) might indicate that introspective processes (e.g., attentional orientation) decrease as extrospective processes increase, and vice versa. Some previous studies also found an anticorrelation between the DMN and the TPN with paranoid patients^28^,^48^,^49^, and normal participants^50^. The reciprocal relationship between the TPN and the DMN has been depicted as low frequency switching between a self-referential, non-task-related introspective state, and an extrospective state that promotes alertness and attentiveness to unexpected or novel events^28^,^49^,^51^. Narcissism is associated with self-focused thoughts and behaviours, which reflect internally-directed cognition^38^,^39^,^40^. The narcissists’ goal is to maintain a positive self-image. However, this fragile self-view is not grounded in an objective reality, so narcissists should constantly seek admiration externally. According to previous studies, females seek their narcissistic goals through more subtle and affiliative means, such as attention seeking (especially in relation to significant others), in order to maintain intimacy and connectedness^11^. The negative correlations between the rGMV of the right SPL and the rsFC between the SPL and the MFG showed that females who had a larger GMV of the SPL had an increased anticorrelation between the DMN and the DAN. This result also indicates that the extrospective process is important in female narcissists. All of these results might suggest that the interplay between externally focused attention and internally focused attention is more dynamic and intrinsic in females with higher narcissism scores.

However, males with higher narcissism scores, had decreased anticorrelations between the right SPL/FEF/insula and areas in the PC, mPFC, and MFG, indicating a lack of balance between DMN and TPN functioning. Previous studies have found an absence of anticorrelated activity between the mPFC and DLPFC in patients with bipolar disorder, schizophrenia^52^,^53^,^54^,^55^, and ADHD^56^,^57^,^58^. Reduced anticorrelations reflect a disparity between the networks, and may arise from dysfunctions in one or both of the networks, most likely indicating aberrant DMN activity^59^. Takeuchi *et al.*^50^ suggested that abnormal DMN activity may distort the boundary between internal thoughts and external perceptions, which may amplify the focus on internal thoughts, and obscure the boundary between internal thoughts and external events. Indeed, to maintain a grandiose self-view, narcissists tend to exaggerate their abilities and experiences, and recount past experiences in self-flattering ways (e.g., taking credit for successes and blaming failure on external causes)^4^,^10^,^12^,^60^,^61^,^62^. Narcissistic males are likely to be excessive in their efforts to assert superiority, being more prone to interpersonal competition than are females^6^,^10^. These males seem to develop blurring of the boundaries between internal reflection and external perception (a grandiose sense of self-importance or uniqueness, with unrealistically high expectations of their acceptance by others), which enhances their narcissism (unrealistic sense of authority and entitlement).

This study has some limitations. One concern is that we collected the data from young healthy subjects with a high level of education. Limited sampling from the full range of the population certainly limits the generalizability of the results^50^,^63^. Previous studies found a negative correlation between age and narcissism^5^. Hence, a much wider range of ages and larger samples will be needed to explore the developmental trajectory of narcissism among males and females in the future. Moreover, we only chose the right SPL as the ROI to perform seed-based functional connectivity analysis to determine the specific brain regions and networks that are related to the sex difference of narcissism. Our analysis can’t exclude some other whole-brain functional connectivity difference between different genders. So future studies can used the data-driven functional connectivity analysis such as independent component analysis to explore some other functional connectivity difference in narcissism between different genders. In addition, some studies have showed that regional and cultural differences appear to exert an influence on narcissism, with participants from more individualistic and independent-oriented societies reporting more narcissism^5^. Our sample, though larger than those previously studied, still only consisted of Chinese participants. As China is deeply influenced by collectivism, the results of our study might only be representative of individuals who grow up in a culture of collectivism. Future studies should examine participants who are influenced by individualistic cultures to explore the neural basis of sex-specific narcissistic personality.

## Conclusion

The present study used a combined structural imaging and rsFC analysis approach to explore the sex-specific neural basis of narcissism in a large healthy sample. In females, higher levels of narcissism were associated with a larger rGMV of the right SPL, and an increased anticorrelation between the SPL/FEF and the PC/MFG (between key nodes of the TPN and the DMN). This indicates that the interplay between externally focused attention and internally focused attention is more dynamic and intrinsic in females with higher narcissism scores. In males, higher narcissism was associated with a decreased anticorrelation between the SPL/FEF and the PC/MFG (between key nodes of the TPN and the DMN), which might reflect a blurring of the boundaries between internal reflection and external perception, which subsequently enhances an unrealistic sense of authority and entitlement. Our results indicate that gender differences in narcissism may be associated with differences in the intrinsic and dynamic interplay between the internally-directed DMN and the externally-directed TPN. In sum, the morphometric and functional connectivity findings can together provide a comprehensive understanding of the neural basis of sex-specific narcissism.

## Methods

### Subjects

This study was conducted as part of an ongoing project to examine the association between brain imaging, creativity, and mental health. In total, 333 healthy right-handed college or postgraduate students were recruited from the local community of Southwest University (China). None of the subjects reported a prior history of neurological or psychiatric disease, or substance abuse.

This study was approved by the local ethics committee of Southwest China University and the Institutional Human Participants Review Board of the Southwest University Imaging Center for Brain Research. The methods were conducted in accordance with approved guidelines. All participants provided written informed consent prior to taking part in the study. The data of three participants were excluded from analysis because of missing answers in the questionnaires. Another four participants were excluded from analysis because of excessive scanner artifacts or abnormal brain structure. Thus, the data of 326 participants, between 18 and 27 years-old were analysed. There were 150 males (mean age = 20.24 years, SD = 1.31) and 176 females (mean age = 19.81 years, SD = 1.29).

### Narcissistic Personality Inventory

The NPI^1^ is a 40-item, forced-choice, self-report measure of narcissism. Although it is based on the clinical criteria for narcissistic personality disorder (as described in the Diagnostic and Statistical Manual of Mental Disorders, version three [DSM-III; American Psychiatric Association, 1980]), it was designed for use with the general population^3^. This scale often is used to assess “normal” or “subclinical” narcissism^1^. The NPI contains 40 pairs of statements, and subjects are asked to choose one statement from each pair that best describes them. Each pair contains a statement that is narcissistic in tone. For example, the statements in one of the pairs are: ‘I am much like everybody else’ and ‘I am an extraordinary person’. Participants score 1 point for each selection of a narcissistic statement. The NPI has seven subscales that measure different dimensions of narcissism: authority, exhibitionism, superiority, entitlement, exploitativeness, self-sufficiency, and vanity. Each subscale consists of three to eight items. Examples of the types of narcissistic statements for each of the seven dimensions are as follows: Authority – ‘I will be a success’; Exhibitionism – ‘I would do almost anything for a dare’; Entitlement – ‘I want to amount to something in the eyes of the world’; Superiority – ‘I think I am a special person’; Exploitativeness – ‘I can usually talk my way out of anything’; Vanity – ‘I like to show off my body’; Self-sufficiency – ‘I always know what I am doing’. The scale has high internal consistency (Cronbach’s α = 0.83), and the inter-correlations among the seven NPI subscales have been reported to range from 0.11 to 0.42^1^. Although the NPI total score was the primary focus of the current study, we also calculated the summary scores of all seven dimensions and performed further analyses on those scores.

### Self-esteem scale

Self-esteem was measured with the 10-item Rosenberg Self-Esteem Scale (SES)^64^,^65^, which is a widely-used instrument with good psychometric properties. Each item is answered on a five-point scale ranging from ‘strongly disagree’ (1) to ‘strongly agree’ (5); a higher score reflects higher self-esteem. Some studies have found self-esteem and narcissism to be related^9^. This questionnaire was used in order to exclude the possibility that a significant correlation between brain structure and NPI was caused by the combination of associations between brain structure and self-esteem, and between NPI and self-esteem.

### Imaging data acquisition

Structural and functional magnetic resonance imaging (MRI) scans were obtained by a 3-T Siemens Magnetom Trio scanner (Siemens Medical, Erlangen, Germany). High-resolution T1-weighted anatomical images were acquired using a magnetization-prepared rapid gradient echo (MPRAGE) sequence (repetition time [TR]/echo time [TE]/inversion time = 1900 ms/2.52 ms/900 ms; flip angle = 9°; slices = 176; slice thickness = 1.0 mm; resolution matrix = 256 × 256; voxel size = 1 × 1 × 1 mm). The whole-brain resting-state functional images were acquired using gradient echo planar imaging sequences, with the following parameters: slices = 32; TR/TE = 2000/30 ms; flip angle = 90°; field of view = 220 mm × 220 mm; thickness slice = 3 mm; slice gap = 1 mm; voxel size = 3.4 × 3.4 × 4 mm^3^.

During resting-state functional magnetic resonance imaging (fMRI) scanning, participants laid supinely with their heads comfortably positioned within the birdcage head coil, which was padded with foam to minimize head movement. Earplugs were used to reduce the influence of scanner noise. All subjects were instructed to relax, keep their eyes closed, and remain still^66^.

### Voxel-based morphometry analysis

The MRI scans were processed using Statistical Parametric Mapping software (SPM8; Wellcome Department of Cognitive Neurology, London, UK [www.fil.ion.ucl.ac.uk/spm/]) implemented in Matlab 7.8 (MathWorks Inc., Natick, MA, USA). MRI scans were first displayed in SPM8 to screen for artifacts or gross anatomical abnormalities. For better registration, the reorientation of the images was manually set to the anterior commissure. The images were segmented into gray matter, white matter, and cerebrospinal fluid using the new segmentation tool in SPM8 toolbox. After segmentation, we performed registration, normalization, and modulation by the Diffeomorphic Anatomical Registration Through Exponentiated Lie algebra (DARTEL) toolbox on SPM8^67^. To ensure that regional differences in the absolute amount of gray matter were conserved, the image intensity of each voxel was modulated by the Jacobian determinants. Finally, the normalized modulated imgaes were smoothed with a 10 mm full-width at half-maximum (FWHM) Gaussian kernel to increase the signal-to-noise ratio.

A voxel-wise analysis of covariance (ANCOVA) was performed to determine whether the relationship between regional gray matter volume (rGMV) and NPI score differed between males and females^68^,^69^. Sex was treated as a condition in the whole-brain analysis. Age, NPI scores, SES scores, and global volumes of gray matter were entered as covariates into the model to control for possible confounding variables. Aside from total brain volume, all covariates were modelled to make the unique relationship of each covariate with rGMV evident for each sex (using the interactions option in SPM8). The interaction effect of sex and NPI scores on rGMV was assessed using t-contrasts. We also tested the interaction effects of sex and each dimension of narcissistic personality (authority, exhibitionism, superiority, entitlement, exploitativeness, self-sufficiency, and vanity) on rGMV.

To avoid edge effects around the borders between gray and white matter, an absolute threshold masking of 0.2 was used. That is, voxels where even a single subject showed the GMV signal intensity of or lower than 0.2 were excluded from the analyses. A correction was performed on the whole-brain data using the voxel-level FWE, and the corrected significance level was set at *P* < 0.05 for all analyses.

### Functional connectivity analysis

The processing of the resting-state image data was performed using the data processing assistant for resting state (DPARSF) software (http://www.restfmri.net/forum/DPARSF)^70^ and the REST toolkit^71^. Both toolboxes were employed using the SPM8 software package. The first 10 volumes of functional images were discarded to account for signal equilibrium and participants’ adaptation to their immediate environment. The remaining 232 images were preprocessed, which included slice timing, head motion correction, and spatial normalization to a standard template. The time courses of various covariates (global signal, white matter, cerebrospinal fluid, and Friston 24-parameter) were extracted and regressed out in order to cancel out the potential impact of physiological artifacts. We used the Friston 24-parameter model to regress out head motion effects from the realigned data based on recent reports that higher-order models can help to reduce head micro movements^72^,^73^. Furthermore, the maximum movement from the original point in each direction was <2 mm. In addition, we included the mean framewise displacement derived from Jenkinson’s relative root mean square algorithm as a nuisance covariate to address the residual effects of motion in group analyses^73^. The images were then resampled to 3-mm cubic voxels. This was followed by spatial smoothing (6 mm FWHM). The smoothed data were linearly detrended and filtered using a band pass filter (0.01 – 0.08 Hz) to eliminate low frequency fluctuations. These functional connectivity preprocessing steps followed the standard protocol reported by Chao-Gan and Yu-Feng^70^.

Functional connectivity was examined using a region of interest (ROI) based method. We are interested in whether the brain areas with sex difference in the GMV are accompanied by sex-specific rsFCs between these regions and other related brain areas. So we chose the brain with sex difference in the GMV as the seed regions to perform seed-based functional connectivity analysis to determine the sex-specific brain regions and networks that are related to narcissism. The significant encephalic region on GMV analysis (a threshold of P < 0.05, corrected for FDR) were defined as ROI for subsequent rsFC analysis, which is the right SPL (peak MNI coordinate, *x, y, z* = 23, −56, 60) and included 41 voxels. To generate the functional connectivity map, the averaged time series was obtained from the ROI, and a correlation analysis was conducted between the ROI and the voxels in the whole brain. The correlation coefficient map was converted into a z-map using Fisher’s r-to-z transformation to improve normalization. For the seed region, the respective individual functional connectivity map z-values were entered into a random effect one-sample t-test in a voxel-wise manner to determine the brain regions that showed significant positive or negative correlations with the seed region. This yielded composite functional connectivity maps, which had a significance level of *P* < 0.05 (FWE corrected) and a cluster size of more than 10 voxels (270 mm^3^).

We then investigated whether the relationship between rsFC and narcissism differed between sex. ANCOVA was used for the whole-brain analysis, wherein sex was a between group factor^74^. Age, mean framewise displacement, NPI scores, and SES scores were entered as covariates into the model to control for possible confounding variables. All of the covariates were modelled so that each covariate’s unique relationship with rsFC within the ROI could be observed for each sex (using the interactions option in SPM8). This facilitated examination of the interaction effect of sex and rsFC covariates. These effects were analyzed using t-contrasts. We also tested the interaction effect of sex and each dimension of narcissistic personality on rsFC, separately. The significant level was set at *P* < 0.05 (FWE corrected).

In addition, we examined sex differences in the correlations between NPI scores and the strength of rsFC in the default mode network (DMN). The seed region was defined as a sphere with a 6-mm radius centered on the peak voxel of the mPFC (−1, 47, −4), and PC/PCC (−5, −49, 40), as reported in previous studies^28^. The same steps were followed as those described above for the functional connectivity analyses.

## Additional Information

**How to cite this article**: Yang, W. *et al.* Gender differences in brain structure and resting-state functional connectivity related to narcissistic personality. *Sci. Rep.* **5**, 10924; doi: 10.1038/srep10924 (2015).

## Figures and Tables

**Table 1:** Demographic data.

	Males (n = 150)			Females (n = 176)		
Measure	Mean	SD	Range	Mean	SD	Range
Age	20.24	1.31	18–26	19.81	1.29	18–27
NPI	14.90	6.37	1–32	12.68	5.96	2–32
SES	39.46	5.62	23–50	39.31	5.08	24–50

Note: SD = standard deviation; NPI = Narcissistic Personality Inventory; SES = Self-Esteem Scale

**Table 2:** The neural correlates of the Narcissistic Personality Inventory.

Seed ROI	Brain regions	H	Peak coordination (MNI)			T score	CorrectedP value (cluster)*	Raw clusterSize (mm^3^)
			X	Y	Z			
Voxel-based morphometry								
/	SPL	R	23	−56	60	5.05	0.017	3120
Functional connectivity								
Right SPL	MFG	R	33	21	21	4.76	0.025	891
Right SPL	Precuneus	R	6	−75	39	5.61	0.000	1809
Right FEF	Precuneus	L	−3	−75	39	4.5	0.002	1206
		R	3	−75	33	5.0		

^*^Corrected at the cluster size threshold with a voxel-level cluster determining a criterion of *P* < 0.001.

Note: ROI = region of interest; MNI = Montreal Neurological Institute; H = hemisphere; L = left; R = right; SPL = superior parietal lobule; MFG = middle frontal gyrus; FEF = frontal eye fields.
